# Polyuridylation in Eukaryotes: A 3′-End Modification Regulating RNA Life

**DOI:** 10.1155/2015/968127

**Published:** 2015-05-11

**Authors:** Paola Munoz-Tello, Lional Rajappa, Sandrine Coquille, Stéphane Thore

**Affiliations:** ^1^Department of Molecular Biology, University of Geneva, 1211 Geneva, Switzerland; ^2^Department of Molecular Therapeutics, The Scripps Research Institute, 110 Scripps Way, Building A Room A265, Jupiter, FL 33458, USA; ^3^University of Bordeaux, European Institute of Chemistry and Biology, ARNA Laboratory, 33607 Pessac, France; ^4^Institut National de la Santé et de la Recherche Médicale, INSERM-U869, ARNA Laboratory, 33000 Bordeaux, France

## Abstract

In eukaryotes, mRNA polyadenylation is a well-known modification that is essential for many aspects of the protein-coding RNAs life cycle. However, modification of the 3′ terminal nucleotide within various RNA molecules is a general and conserved process that broadly modulates RNA function in all kingdoms of life. Numerous types of modifications have been characterized, which are generally specific for a given type of RNA such as the CCA addition found in tRNAs. In recent years, the addition of nontemplated uridine nucleotides or uridylation has been shown to occur in various types of RNA molecules and in various cellular compartments with significantly different outcomes. Indeed, uridylation is able to alter RNA half-life both in positive and in negative ways, highlighting the importance of the enzymes in charge of performing this modification. The present review aims at summarizing the current knowledge on the various processes leading to RNA 3′-end uridylation and on their potential impacts in various diseases.

## 1. Introduction

RNA 3′-end processing or modification plays an important role in determining their biological fate [1–3]. One major type of modification encountered by mRNAs is the addition of nontemplated nucleotides [3–7]. The main functional consequence of this nucleotide addition is to protect newly transcribed mRNAs from degradation. More generally, tail addition to RNAs regulates cellular RNA content by influencing RNA steady-state levels. Nuclear polyadenylation is essential to degrade various classes of noncoding RNAs (ncRNAs) in the nucleus [8–11]. However, once in the cytoplasm, RNAs carrying a 3′-poly(A) tail are protected from 3′ to 5′ exonucleases. Polyuridylation is another 3′ modification that involves the addition of uridines at the 3′-end of RNA molecules. This modification is found on various types of RNAs such as mRNAs, small RNAs, miRNAs, or guide RNAs (gRNAs) [7, 12–22]. This modification is known to have a major impact in multiple aspects of RNA turnover and metabolism, which are reviewed hereafter [7, 13–15, 20, 21].

### 1.1. Polyadenylation

Eukaryotic mRNAs start to be modified during their transcription, where capping and polyadenylation take place at their 5′- and 3′-ends, respectively, except for histone and some viral mRNAs [23]. Pre-mRNAs are first cleaved by the cleavage and polyadenylation machinery at the polyadenylation site located near the potential 3′-end. This cleavage is followed by the addition of the poly(A) tail by nuclear poly(A) polymerases (PAPs). This event will determine the 3′ untranslated region (UTR) of the RNA, which is crucial for the regulation of gene expression processes [24]. Mutations and changes in the length of this region will immediately affect a variety of processes such as mRNA stability, mRNA localization, and mRNA translation efficiency [25–29]. Once the mRNAs are exported to the cytoplasm, they may undergo several additional modifications such as methylation, editing, deadenylation, decapping, and polyuridylation, which again influence the stability or degradation of the RNA [7, 14, 17, 20–22, 30–35]. Polyadenylation regulates RNA degradation, which is one of the most important gene expression mechanisms not only for the removal of mRNAs that should not be translated anymore, but also for the disposal of the incorrectly transcribed mRNAs that have escaped the nuclear surveillance mechanisms. The general basis of RNA degradation is well conserved throughout eukaryotes, from yeast to mammals, and has two major directions: the 5′-3′ degradation by Xrn1 exoribonuclease and the 3′-5′ degradation catalyzed by the exosome complex (for recent review, see [36]). However, before degrading the mRNA bodies, cells must first identify the mRNAs to degrade. The cellular cues initiating mRNA degradation are still poorly understood for mRNAs encoded by the so-called “house-keeping” genes, while physiological inputs that trigger mRNA decay such as proinflammatory responses, heat shock, or differentiation are far better characterized [37, 38]. Deadenylation is generally the rate-limiting event in the cytoplasmic mRNA degradation and is catalyzed by the PAN2/PAN3 complex followed by the CCR4/NOT complex [31, 35]. Once the poly(A) tail has been removed, the Dcp1-Dcp2 decapping complex will withdraw the 7-methylguanylate cap from the 5′-end of the mRNA allowing the trimming of this RNA in a 5′ to 3′ manner by Xrn1 exonuclease [31–33, 39, 40]. Following deadenylation, the cytoplasmic exosome complex may cut down deadenylated RNAs as the 3′-5′ mRNA decay pathway [41, 42].

### 1.2. Polyuridylation

Recently, another player in the mRNA decay pathways has come into focus: the cytoplasmic poly(U) polymerases. These enzymes add uridine residues to the 3′-end of either coding RNAs or ncRNAs. Even though this modification has been known since the late fifties, its significance had been underestimated [43–45]. In the middle of the eighties, the importance of uridylation increased with the discovery and the characterization of the uridine insertion/deletion editing mechanisms in the mitochondria of kinetoplastids. This process was subsequently shown to be crucial for generating functional mRNA sequences as well as for increasing translation efficiency of local mRNAs [14, 30, 34]. Studies from the Aphasizhev laboratory on poly(U) polymerase family members present in trypanosomal species demonstrated additional roles for these enzymes, not only in the uridine insertion/deletion mechanism (generally known as the RNA editing process) but also during the processing of gRNA molecules and during mitochondrial mRNA translation [46, 47]. During the last decade, evidence showed that polyuridylation also existed in higher eukaryotes. The team of C. Norbury was the first to show that cells overexpressing a cytoplasmic poly(U) polymerase named Cid1 were less sensitive to hydroxyurea treatment, although the exact molecular mechanism was not fully understood [48]. Further studies demonstrated that polyuridylation was a critical step for the degradation of nonpolyadenylated mRNAs encoding histone proteins in mammals [20]. This new enzymatic step occurring at the 3′-end of nonpolyadenylated and polyadenylated mRNAs added another level of complexity to the known mRNA decay pathways [7, 20, 21, 49]. Finally, polyuridylation has also been found to occur in other types of RNA molecules such as miRNAs, siRNAs, and piRNAs with various functional consequences described hereafter [12, 16–19, 22].

In this review, we focus on the latest research about the terminal polyuridylation by a specific group of noncanonical ribonucleotidyl transferases, a long time underestimated 3′-end posttranscriptional modification found in various RNAs and influencing RNA half-life and functions. The review will be divided in the following sections including a brief overview of the nucleotidyl transferase family followed by a review of the functional consequences of RNA polyuridylation in the different cell compartments. Finally, we will touch upon the multiple implications of polyuridylation mechanisms in diseases.

## 2. The Noncanonical Ribonucleotidyl Transferase Family

Enzymes performing terminal polyuridylation belong to the polymerase *β*- (Pol *β*-) like nucleotidyl transferase superfamily and more specifically to the group of template-independent polymerases that covalently add nucleotides to the 3′-end of RNA molecules. This protein family was precisely defined a few years ago [5]. Briefly, proteins from this family are named RNA-specific nucleotidyl transferases (rNTrs) and classified in three subgroups: (i) The canonical group, which corresponds to the nuclear poly(A) polymerases *α*, *β*, and *γ*. These are found in eukaryotes and share similar enzymatic and RNA-binding domains. (ii) The noncanonical rNTrs regroup a variety of proteins such as the Gld-2-, Trf4/5- and Cid1-type of poly(A) or poly(U) polymerases, the 2′-5′-oligo(A) synthetases, and the trypanosomal terminal uridylyl transferases. (iii) The third group is the one of the CCA-adding enzymes. In this review, we will only focus on the noncanonical rNTrs group as previously defined in [5].

Every member from the noncanonical rNTrs group is characterized by an enzymatic domain made of two lobes named the catalytic and the central domains. The catalytic domain is made of four or five *β*-strands. The second *β*-strand contains a DxD or DxE motif (aspartate “D” or glutamate “E” residues separated by one hydrophobic residue “x”). A third aspartic residue is found in the third *β*-strand of the catalytic domain. The catalytic reaction is similar to the one described for the Pol *β* enzyme that includes a nucleophilic attack on the alpha phosphate of the bound nucleotide triphosphate by the 3′-OH group of the RNA substrate. The three aspartate residues interact with the incoming RNA and two metal ions necessary to stabilize the reaction intermediate as described previously [50]. The central domain contains the nucleotide recognition motif (NRM), which corresponds to a 10–15 amino acid long loop forming one end of the nucleotide triphosphate binding pocket. The residues located in the NRM stabilize the base of the substrate nucleotide triphosphate via water-mediated and/or direct hydrogen bonds with their side chain atoms [51–53]. Subclassification of the rNTrs was attempted based on the local amino acid sequence conservation of the NRM. However, in light of recent crystal structures from members of the rNTrs in complex with their natural substrate NTP, it appears that using the NRM sequence identity may not be sufficient to precisely predict the type of nucleotide accepted in the active site. In fact, it is not fully clear whether these proteins are not able to add various types of nucleotide* in vivo* as recent sequencing studies specifically designed to identify 3′-addition of nontemplated nucleotides highlighted the diversity of the cellular RNA tails [54].

Furthermore, a RNA recognition motif (RRM) is also found in all canonical and a few noncanonical rNTrs. Its likely role is to bind RNA substrates in a non-sequence-specific manner [55, 56]. The RRM domains are differentially located in the sequence, that is, near the C-terminus for the canonical PAPs, at the N-terminus, or in the central domain in some noncanonical rNTrs. The RBD is absent in numerous noncanonical rNTrs enzymes, indicating that either these proteins can act on any RNA or that their activity is restricted via a protein partner that targets them to specific RNAs or both. In at least one case, the enzyme ZCCHC11 is targeted to one specific pre-miRNA species through interactions with the Lin28 proteins [57–59].

From a phylogenetic point of view, several models have been proposed to explain the evolution of the Pol *β*-NTrs family. The hypothesis of Aravind and Koonin [60] is that the Pol *β*-NTrs family members have rapidly and independently diverged from a common ancestor presenting a very general and nonspecific nucleotidyl transferase activity. The different family members would have acquired distinct functional domains to occupy vacant evolutionary niches. Then, horizontal gene transfer and lineage-specific gene loss could have explained the actual distribution of the different groups in the three domains of life. Some evidences like the discovery of the archaeal and bacterial minimal nucleotidyl transferases (MNT family) and the restricted phylogenetic distribution of most of the Pol *β*-NTrs family members support this model [60]. However, it has recently been shown that a bacterial poly(A) polymerase that possesses the RBD of a CCA-adding enzyme is able to act as a CCA-rNTrs [61]. This suggests that the CCA-adding enzymes could be the ancestors of the poly(A) polymerases and possibly the founders of all the remaining rNTrs, which would have adopted different RNA binding domains mediating different target specificity.

The noncanonical rNTrs is divided into two main groups based on their specific activities: the Cid1-like family and the RNA editing enzymes.

(i) The Caffeine-induced death suppressor protein 1 (or Cid1) from* Schizosaccharomyces pombe* is the pioneer of cytoplasmic poly(U) polymerases [62]. Many other proteins are part of this group with highly similar enzymatic properties but limited sequence homology such as the trypanosomal protein RNA editing TUTase1 (RET1). Despite its name, RET1 modifies specifically the 3′-end of both the gRNAs and the mRNAs in kinetoplast without any involvement in the RNA editing process itself [13, 15, 46, 47, 63]. Seven proteins from this group are found in human. Evidences start to accumulate for some of these human proteins but, globally, their precise action still requires a more detailed characterization [58, 62, 64].

(ii) The RNA editing enzymes, on the other hand, are responsible for mitochondrial mRNA editing by U-insertion/deletion [65–70]. Mainly, two proteins have been studied extensively: RNA editing TUTase 2 (RET2) and the mitochondrial editosome-like complex associated* TUTase 1* (MEAT1). RET2 and MEAT1 are found with the 20S editosome complex of trypanosomes and are crucial for the U insertion-type of editing in this organism [70, 71]. Crystal structures of RET2 and MEAT1 showed a conserved domain organization except for the middle domain [51, 52]. The lack of sequence similarity within this middle domain suggests divergent functions.

## 3. Polyuridylation according to Cell Compartments

**Figure 1:**
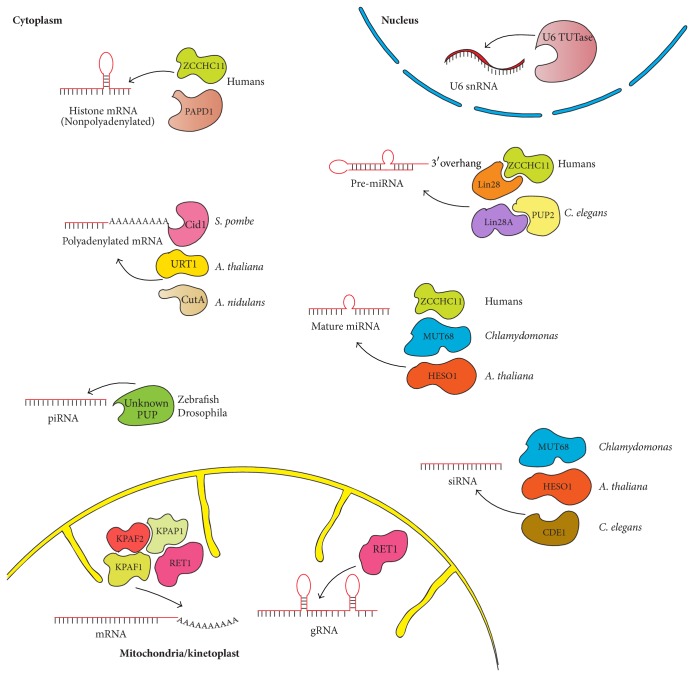
Substrates of polyuridylation in the different cell compartments. For each substrate, the players for polyuridylation are presented for the organisms mentioned. The curved arrows illustrate the polyuridylation event.

Until a few years ago, polyuridylation had been only reported in the mitochondria of the parasitic protist trypanosome [14, 30]. More recently, noncanonical rNTrs were found in the cytoplasm of various eukaryotic species and were shown to modify a wide range of nontranslated and translated RNAs [7, 17, 20, 21, 72–75]. Details of the different substrates and the responsible enzymes in the cell nucleus, cytoplasm, and mitochondria are described hereafter and summarized in Figure 1.

### 3.1. In the Nucleus

**Figure 2:**
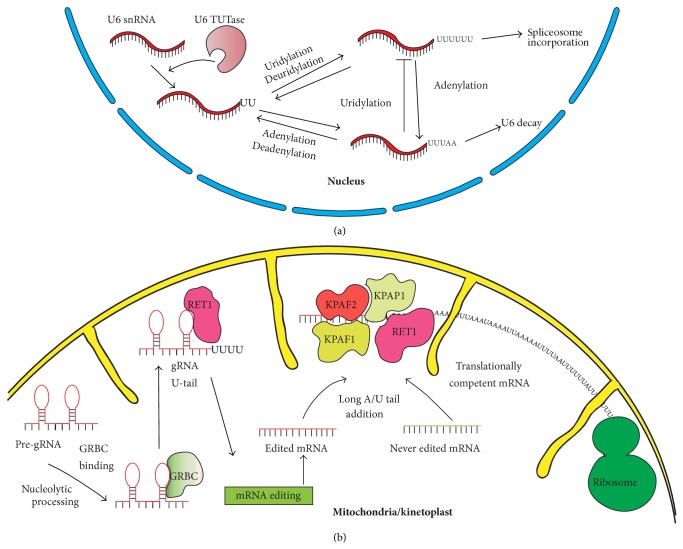
Known functions of polyuridylation in the nucleus and in the mitochondria. (a) Functions in the nucleus. U6 snRNA is the only known substrate for polyuridylation in the nucleus by U6 TUTase. Polyuridylation is thought to regenerate the 3′-end of U6 snRNA following its shortening by exonucleases. If this RNA is adenylated, the polyuridylation event is inhibited and the U6 snRNA is degraded. If the RNA is polyuridylated, mature U6 snRNA is produced and incorporated in the splicing complex known as the spliceosome. (b) Function of polyuridylation in the mitochondria of trypanosomes. In order to be properly matured, gRNAs are polyuridylated by RET1 TUTase allowing the gRNA to “guide” the editing reaction. To be translationally competent, mitochondrial mRNAs require addition of a long A/U tail, which is performed by the RET1/KPAP1 complex and coordinated by the KPAF1/KPAF2 complex. The mRNA is then recognized by the ribosome and translation can be started.

Until now, the only substrate of uridylation reported in the nucleus is the U6 snRNA (Figure 1). This RNA is uridylated by the U6 TUTase, which is an essential enzyme for cell survival in mammals [64]. SiRNA-mediated silencing of the U6 TUTase leads to U6 snRNA decay, confirming the necessity of uridylation for U6 snRNA stability [64]. U6 TUTase is responsible for the addition or restoration of at least four uridine residues at the 3′-end of U6 snRNA since 3′-end of U6 snRNA is constantly subjected to exonucleases activity [64, 76]. These four U residues form an intramolecular double strand with a stretch of adenines within the U6 snRNA molecule, which is important for mRNA splicing [64]. This uridylation event specifically in the nucleus allows the proper production of a splicing-competent U6 snRNP (Figure 2(a)). Mammalian U6 snRNA uridylation* in vivo* has been reported with up to 20 nucleotides added at the 3′-end of the RNA molecule [77, 78]. It is important to note that U6 snRNA is also subjected to adenylation and this event inhibits its uridylation (Figure 2(a)) [79]. Moreover, the 3′-end of U6 snRNA is recognized specifically by the Lsm2-8 complex, a doughnut-like heteroheptameric complex related to the Sm complex found on the snRNPs.

### 3.2. In the Organelles (Mitochondria)

Uridylation events in the organelles have been reported in mitochondria [13, 63]. So far, no polyuridylation events have been found in the chloroplast of plants and algal cells. It is apparently absent, although proteins from the rNTrs family are present such as the poly(A) polymerase [80]. One possible reason is the close evolutionary conservation of the RNA processing pathways found in the chloroplast and in bacteria where poly(A) tail present at the 3′-end of mRNAs is the major regulatory modification [81–84].

Poly(U) tails have been reported mostly in kinetoplastid-containing organisms. Two main substrates are targeted in these organisms: gRNAs and locally transcribed mRNAs (Figure 1). The gRNAs are specific to kinetoplastid species and are crucial for cell survival as they are in charge of guiding the RNA editing machinery to its mRNA targets [14]. RET1, the first characterized ncNTrs, acts on both types of RNAs with strikingly different functional consequences.

For the gRNAs, uridylation represents their final maturation step [13, 46]. In order to be matured, pre-gRNAs need to pass through an exonucleolytic process followed by stabilization by the gRNA binding complex (GRBC) and RET1 uridylation (Figure 2(b)) [13, 15, 85]. Mature gRNA is thus composed of a 5′ phosphate from the transcription followed by an anchor region complementary to a target unedited mRNA, a guiding region that directs the editing of its mRNA target and a final poly(U) tract at the 3′-end. In RET1-depleted cells, gRNAs are stable but not able to perform their editing function suggesting a crucial role of the oligo(U) tail in the editing event in the mitochondria. This oligo(U) tract may stabilize the gRNA-mRNA hybrid through binding with the purine-rich preedited region [15]. The uridylated gRNA bound to its mRNA target recruits the 20S editosome. This gRNA-mediated mRNA editing in kinetoplastid trypanosomes is crucial for the parasite survival, as these editing events are needed for the proper establishment of the coding sequence of the mitochondrial mRNAs [15]. Currently, it is not yet fully understood how RET1 enzyme recognizes its gRNA substrate nor how the pre-gRNA processing step takes place [13, 46].

After editing, mRNAs need to be further modified at the 3′-end in order to be translationally competent in trypanosomal mitochondria. This modification is the addition of a long 3′ A/U tail (Figure 2(b)) [47]. This nucleotide addition is due to RET1 which works in concert with the kinetoplast poly(A) polymerase 1 (KPAP1). The RET1/KPAP1 complex adds approximately 200 alternated adenines and uridines to the 3′-end of the targeted mRNAs [47]. Therefore, polyuridylation and polyadenylation are necessary to trigger the translation of edited as well as never edited mRNAs (Figure 2(b)). RET1 and KPAP1 actions are coordinated by the kinetoplast polyadenylation/uridylation factors 1 and 2 (KPAF1 and KPAF2) complex [47]. Currently, our molecular understanding of the sequence of events taking place at the 3′-end of mitochondrial mRNAs is poor and awaits further structural and biochemical characterization [47, 63].

It is noteworthy that RNAs with poly(U) tails have also been observed in human mitochondria under certain conditions [86–89]. In spite of this, how this process is achieved in this compartment and its implication(s) for human mitochondrial RNA metabolism still remain to be characterized.

### 3.3. Polyuridylation in the Cytoplasm

**Figure 3:**
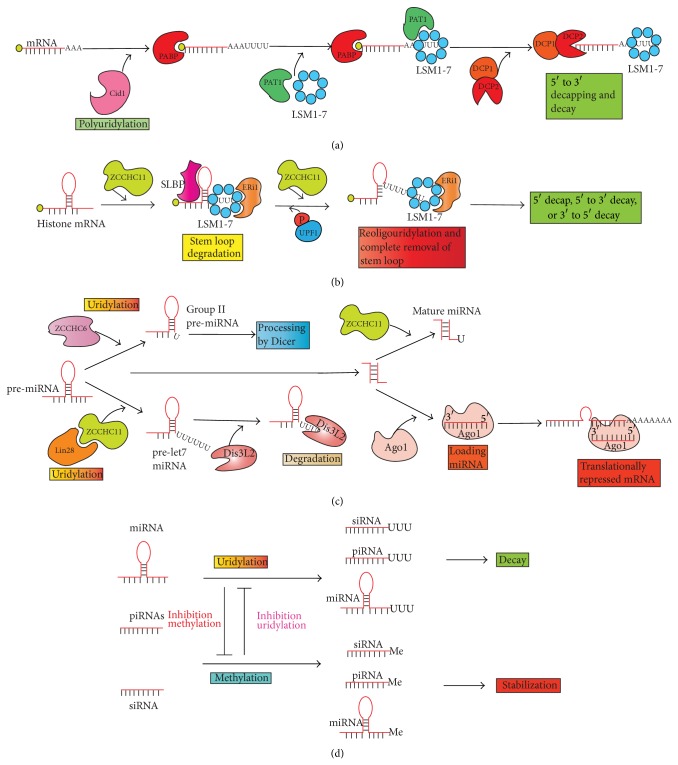
Known functions of polyuridylation in the cytoplasm. (a) In* S. pombe*, polyuridylation of mRNAs by Cid1 PUP leads to decapping and decay. (b) In* Humans*, histone mRNAs are uridylated by ZCCHC11 followed by LSM1-7 complex binding. ERI1 enzyme will then bind the LSM1-7 complex inducing the histone mRNA stem loop. The stem loop degradation will stall and reoligouridylation and possibly UPF1 helicase activity are needed in order to proceed. The remains of the stem loop are degraded leading to histone mRNA decay from the 5′ and/or 3′-end. The exact moment when SLBP (stem loop binding protein) is released during mRNA decay is currently unknown. (c) In* Humans*, ZCCHC11 in concert with Lin28 polyuridylates pre-let7-miRNA, which will then be degraded by DIS3L2 exonuclease. ZCCHC6 alone is responsible for the monouridylation of group II pre-miRNA, which will be further processed by Dicer. In the case of mature miRNA, ZCCHC11 monouridylates some miRNA leading to indirect consequences to the miRNA targeted mRNAs. (d) In plants, zebrafish, and flies, methylation and polyuridylation have antagonistic effects. Methylated siRNAs, piRNAs, and miRNAs will be stabilized whereas the polyuridylated one will be degraded.

Cytoplasmic polyuridylation occurs on a variety of RNA molecules ranging from polyadenylated to nonpolyadenylated RNA molecules including mRNAs, small RNAs, miRNAs, or piRNAs (Figure 1) [7, 17, 20, 21, 73–75]. The various functional outcomes of polyuridylation in this compartment offer new insights into RNA turnover and small RNA biogenesis (Figure 3).

Several eukaryotic mRNAs were shown to be uridylated in the cytoplasm of* S. pombe* by the poly(U) polymerase Cid1 (Figure 1) [7, 62]. RNA cRACE studies in fission yeast revealed a role of uridylation in a new deadenylation-independent decapping-mediated degradation pathway (Figure 3(a)) [7]. Until now, only a handful of mRNAs has been identified to be specifically uridylated such as* act1, urg1,* and* adh1* [7]. Recent studies looked at the 3′-end sequence of mRNAs at a genome-wide level and revealed that U tails are apparently attached to short poly(A) tracks rather than to the mRNA body [49, 54]. Interestingly, while some mRNAs like the one encoding the poly(A) binding protein 4 are polyuridylated in more than 25% of the cases, about 80% of mRNAs have an uridylation frequency comprised between 2 and 5%. Overall, the functional relevance of those low-level of uridylation is currently unknown. Factors such as ZCCHC6 or ZCCHC11 (also known as TUT7 and TUT4 resp.) have been shown to be responsible for the human cytoplasmic mRNA uridylation activity and the consequence is apparently to induce mRNA degradation [49]. Furthermore, a single uridine at the 3′-end of a RNA molecule is sufficient to be recognized by the Lsm1-7 complex, known to link 3′-end deadenylation and 5′-end decapping, clearly supporting the relationship between uridylation and mRNA degradation [90].

Nonpolyadenylated mRNAs are also uridylated in the cytoplasm (Figure 1). This is the case of the histone-encoding mRNAs [20]. Upon inhibition of DNA replication or conclusion of S-phase, histone proteins are not necessary anymore and, so, histone mRNAs must be rapidly degraded in order to avoid their accumulation and their interference with other cellular pathways [91]. Histone mRNAs are not polyadenylated but possess a stem loop structure at their 3′-end crucial for pre-mRNA processing, export, and proper translation [92–94]. Studies aiming to understand the mechanism by which histone mRNA degradation was triggered found that histone mRNAs were targeted to decay by uridylation (Figure 3(b)) [20, 21]. The nature of the responsible enzyme(s) is still the subject of conflicting results as different groups found different enzymes [20, 21]. These studies systematically found Cid1 orthologous enzymes such as TUTase1 (PAPD1), TUTase3 (Trf4-2), and ZCCHC11 (TUT4) to be responsible for the uridylation. It is not fully clear; however, how PAPD1 enzyme would either switch between cell compartments as PAPD1 is reported as a mitochondrial protein or how they could select which substrates to uridylate and which one to adenylate* in vivo* as both PAPD1 and Trf4-2 proteins do have reported poly(A) polymerizing activities [20]. One could not exclude that several pools of PAPD1 differentially located in the cell may exist. More data are definitely required to fully apprehend rNTrs role during regulated histone mRNA degradation in particular regarding the factors bringing together the histone mRNAs and the rNTrs. Interestingly, as for polyadenylated mRNAs in fission yeast, uridylation of histone mRNAs was shown to promote decapping followed by 5′-3′ degradation [20]. The Lsm1-7 protein complex was shown to be responsible for the promotion of the decapping activity. More recently, 3′-5′ degradation of histone mRNAs by the exonuclease ERI1 has been reported (Figure 3(b)) [95]. Again, the Lsm1-7 complex was involved in the recruitment of the exonuclease ERI1 to the terminal stem loop. The Lsm1-7 complex apparently binds both the uridylated histone mRNAs and the exonuclease ERI1 [95].

A variety of ncRNAs from diverse organisms have recently been shown to carry mono- or multiple non-templated uridine residues at their 3′-end (Figure 1) [19, 73, 96–98]. The major functional consequence associated with uridylation is to trigger RNA degradation but is not limited to it. 3′ uridylation of various miRNAs has been observed in multiple sequencing studies suggesting a wide role of uridylation during miRNA biogenesis [99–101]. Mono- or polyuridylation events have been found in both pre-miRNAs and mature miRNAs [73, 96, 98, 102]. In* C. elegans* and* H. sapiens*, polyuridylation of pre-let-7-miRNA has been reported and is performed by the proteins PUP-2 and ZCCHC11, respectively [98, 103, 104]. Association between the pre-miRNA and the Lin28 protein induces a conformational change in the pre-miRNA loop, which possibly favors modification by ZCCHC11 [105, 106]. However, the presence of a single 3′-overhanging nucleotide appears critical for the uridylation process therefore excluding the so-called “group I” or canonical miRNAs from being subject to uridylation [96]. Furthermore, in the same study, ZCCHC6 enzyme was found to be responsible for the monouridylation of group II let-7 pre-miRNAs and this modification is independent of the Lin28 protein but is critical for the production of this particular miRNA [96]. So, uridylation of pre-miRNAs can influence the miRNA production both positively and negatively (Figure 3(c)) [21]. Finally, ZCCHC11 has also been involved in the uridylation of specific mature miRNA such as miR-26 [107]. Further biochemical and biophysical studies are needed in order to identify the specific enzymes responsible for the uridylation of other miRNAs in higher organisms as well as the target-specific effects induced by this 3′-end modification. Interestingly, mammalian Dis3L2 exonuclease was also shown to specifically degrade uridylated pre-let-7-microRNA discriminating them from 3′-unmodified RNAs [108]. Recently, Dis3L2 protein was shown to preferentially degrade mRNAs with 3′-end uridylation and its deletion together with the one of Lsm1 led to the accumulation of uridylated mRNAs in fission yeast (Figures 3(a) and 3(c)) [109]. Further studies between the Dis3L2 exonuclease and TUTases will be necessary to better understand their respective functions and the link existing between these enzymatic activities.

Other types of ncRNAs subject to 3′ uridylation are siRNA and piRNAs (Figure 1). In nematodes and in plants, these particular types of RNA substrate are modified by the protein CDE-1 (cosuppression defective 1) and HESO-1 (Hen1 suppressor1) respectively [110]. In the green algae Chlamydomonas, MUT68 has been implicated in this event [73]. Studies in plant and animal species have demonstrated an antagonistic role of uridylation and 2′-*O*-methylation in these organisms [12, 17–19, 22]: Hen1 (HUA ENHANCER 1) and its homologs methylate sRNAs in plants, piRNAs in vertebrates, and Ago2-associated siRNAs in flies, protecting these RNAs against 3′ uridylation (Figure 3(d)) [12, 17–19, 22]. In* C. elegans*, CSR-1 is an Ago protein necessary for proper chromosome segregation rather than regulation of mRNA levels [74, 111, 112]. CDE-1, a* C. elegans* PUP, uridylates unmethylated siRNAs of the CSR-1 pathway [74]. Mutation of this CDE-1 enzyme leads to accumulation of CSR-1 siRNAs, which promotes erroneous chromosome segregation and defective gene silencing [74]. Uridylation is then a destabilizing factor against CSR-1 siRNAs, which regulates CSR-1-dependent and specific siRNA levels in this organism. In* Chlamydomonas reinhardtii*, MUT68 was first known to adenylate 5′ cleavage fragments of mRNAs targeted by the RNA-induced silencing complexes (RISC), thereby promoting their decay [97]. Further studies showed an important role of MUT68 in miRNA and siRNA degradation through its 3′ uridylation activity [73]. 3′ uridylation of piRNAs have been observed in zebrafish and drosophila, but the enzymes responsible for this modification are currently unknown [12, 17]. In zebrafish Hen1 mutants, piRNAs derived from retrotransposons are found uridylated and their levels are decreased suggesting a sensitivity of these uridylated piRNAs to degradation. Interestingly, a mild repression of retransposons is observed in these mutants thus highlighting a destabilizing role for uridylation of piRNAs and a stabilizing role for methylation [17]. Taken together, these data highlight the crucial roles of small ncRNA uridylation within diverse biological processes and in several organisms. Defects in the regulation of this phenomenon can have important consequences on gene expression (Figures 2 and 3(d)).

The HESO-1 enzyme, like MUT68, is also shown to act on atypical substrates, that is, the product of the miRNA-directed mRNA cleavage [113]. In this case, the uridine nucleotides are apparently added to the 5′-fragment of the cleaved mRNA when it is still bound by the Ago1 complex [113]. Further studies will help determining the generality of this mechanism as HESO-1 does not seem to be conserved in higher eukaryotes.

At last, polyuridylation has also been reported to stabilize RNAs, rather than destabilize them. In* Arabidopsis thaliana*, uridylation of oligoadenylated mRNAs has been suggested to prevent their 3′ trimming and rather establish a preferential 5′-to-3′ mRNA degradation manner [114]. Indeed, URT1 (UTP:RNA uridylyl transferase 1) was shown to uridylate oligo(A)-tailed mRNAs* in vivo* and its absence contributed to the degradation of oligoadenylated mRNAs highlighting a new role of uridylation in mRNA stabilization. The ZCCHC11 enzyme, besides its role in histone mRNA and pre-miRNA decay, has also been implicated in indirect mRNA stabilization by uridylation of mature miRNAs (Figure 3(c)). ZCCHC11-dependent uridylation of mature cytokine-targeting miRNAs is known to lead to the stabilization of cytokine transcripts and hence regulates cytokines gene expression. Mature miR-26 can bind interleukin IL-6 mRNA in its 3′ UTR and targets this cytokine-encoding mRNA to degradation [107]. Upon miR-26 uridylation by ZCCHC11, the miRNA is unable to bind the 3′ UTR of the mRNA and thus the transcript is stabilized with no associated degradation of miR-26. This is further confirmed by ZCCHC11 knockdown experiments where several cytokine mRNAs are downregulated in the absence of uridylation [107, 115].

These data together support a crucial role of cytoplasmic polyuridylation in the regulation of gene expression and stability control of both coding and noncoding RNAs in diverse eukaryotic species.

## 4. Polyuridylation and Diseases

RNA uridylation in the cytoplasm has been shown to induce tumorogenesis in mammals. Uridylation at the 3′-end of the tumor suppressor pre-let-7 microRNA by cytoplasmic ZCCHC11 and ZCCHC6 enzymes blocks let-7 miRNA maturation, which in turn stimulates tumor growth [58]. Lin28 is a factor of pluripotency in stem cells and once it is expressed, it helps the maintenance of an undifferentiated and proliferative state by blocking the expression let-7 miRNA by recruiting ZCCHC11 for uridylation-mediated decay [57–59]. In adult somatic cells, Lin28-let-7 pathway is normally silenced even though we still observe expression of LIN28A or LIN28B in a wide variety of human cancers [116, 117]. Inhibition of this oncogenic pathway blocks the tumorigenicity of cancer cells [116]. It has recently been shown that modified let-7 microRNAs are degraded by Dis3L2 exonuclease [118]. Furthermore, Dis3L2, which preferentially trims uridylated cytoplasmic RNAs, has been found mutated in patients with Perlman syndrome and in some cases this mutation lead to the development of Wilm's tumor at early stages of child's growth [118]. Even though RNA uridylation has been linked to tumor growth, the biological significance of such event is still poorly understood and as such is being studied. In order to better understand tumorigenesis, it is necessary to identify the RNA targets as well as the protein partners that recruit either the RNA substrates or the poly(U) polymerases. Such information will allow the in-depth studies of the link between PUPs and diseases. Furthermore, structural and biochemical studies of substrate recognition by rNTrs will provide a rational foundation for therapeutic purposes. In kinetoplastid organisms, this information will bring new insights into U-insertion/deletion, gRNA biogenesis, and translational control required for parasite survival. Thus, it may provide a new avenue for the design of new trypanocides, important to treat various trypanosomal diseases including the fatal human sleeping sickness.

## 5. Conclusions and Perspectives on Polyuridylation

Polyuridylation was for a long time an underestimated 3′-end modification; most probably because sequencing techniques were focused on polyadenylated RNAs. With the development of new and adapted techniques to detect 3′ uridylation, this event is starting to gain strength with impacting roles in RNA degradation and stability [99, 119]. RNA sequencing analysis of mammalian cells, not depending on oligodT primers but rather using 3′ ligated linkers specific for small RNAs of 200 nt or less, showed a widespread tendency of 3′-end uridylation of small RNAs [99]. Interestingly, besides the already known uridylated targets, they also found this 3′ modification on transcriptional start-site-associated RNAs along with spliced introns. This suggests a larger role of polyuridylation in RNA metabolism in mammals, despite the fact that PUPs are mostly localized in the cytoplasm. Optimized RNA sequencing methods in different backgrounds, such as DNA replication inhibition and stress conditions, and refinements in these methods, are necessary to understand the global biological consequences of uridylation in RNA metabolism. With RNA-Seq development, more and more RNAs are found to be uridylated in various organisms, but the enzymes responsible for this process are still unknown. The identification of polyuridylating enzymes becomes now critical for obtaining a larger picture of uridine tail addition in eukaryotes, its evolution, and its functional implication in the cell. Finally, 3′ uridylation is involved in several key aspects of RNA biology and all the proteins implicated in this process in eukaryotes are not yet known. It thus brings into focus the importance of multiplying studies concerning this particular process and the relevant players. Several research groups nowadays started to focus their work on identifying new rNTrs along with their targets and possible protein partners. We will most likely hear a lot more about rNTrs and their influence on RNA metabolism and turnover during the coming years.

## References

[1] Alexandrov A., Chernyakov I., Gu W. (2006). Rapid tRNA decay can result from lack of nonessential modifications. *Molecular Cell*.

[2] Meyer S., Temme C., Wahle E. (2004). Messenger RNA turnover in eukaryotes: pathways and enzymes. *Critical Reviews in Biochemistry and Molecular Biology*.

[3] Munroe D., Jacobson A. (1990). mRNA poly(A) tail, a 3′ enhancer of translational initiation. *Molecular and Cellular Biology*.

[4] Doma M. K., Parker R. (2007). RNA quality control in eukaryotes. *Cell*.

[5] Martin G., Keller W. (2007). RNA-specific ribonucleotidyl transferases. *RNA*.

[6] West S., Gromak N., Norbury C. J., Proudfoot N. J. (2006). Adenylation and exosome-mediated degradation of cotranscriptionally cleaved pre-messenger RNA in human cells. *Molecular Cell*.

[7] Rissland O. S., Norbury C. J. (2009). Decapping is preceded by 3′ uridylation in a novel pathway of bulk mRNA turnover. *Nature Structural and Molecular Biology*.

[8] Deutscher M. P. (1990). Ribonucleases, †RNA nucleotidyltransferase, and the 3′ processing of †RNA. *Progress in Nucleic Acid Research and Molecular Biology*.

[9] Keller W. (1995). No end yet to messenger RNA 3′ processing!. *Cell*.

[10] LaCava J., Houseley J., Saveanu C. (2005). RNA degradation by the exosome is promoted by a nuclear polyadenylation complex. *Cell*.

[11] Manley J. L. (1995). Messenger RNA polyadenylylation: a universal modification. *Proceedings of the National Academy of Sciences of the United States of America*.

[12] Ameres S. L., Horwich M. D., Hung J.-H. (2010). Target RNA-directed trimming and tailing of small silencing RNAs. *Science*.

[13] Aphasizheva I., Aphasizhev R. (2010). RET1-catalyzed uridylylation shapes the mitochondrial transcriptome in *Trypanosoma brucei*. *Molecular and Cellular Biology*.

[14] Blum B., Bakalara N., Simpson L. (1990). A model for RNA editing in kinetoplastid mitochondria: ‘Guide’ RNA molecules transcribed from maxicircle DNA provide the edited information. *Cell*.

[15] Blum B., Simpson L. (1990). Guide RNAs in kinetoplastid mitochondria have a nonencoded 3′ oligo(U) tail involved in recognition of the preedited region. *Cell*.

[16] Horwich M. D., Li C., Matranga C. (2007). The *Drosophila* RNA methyltransferase, DmHen1, modifies germline piRNAs and single-stranded siRNAs in RISC. *Current Biology*.

[17] Kamminga L. M., Luteijn M. J., Den Broeder M. J. (2010). Hen1 is required for oocyte development and piRNA stability in zebrafish. *EMBO Journal*.

[18] Kurth H. M., Mochizuki K. (2009). 2′-O-methylation stabilizes Piwi-associated small RNAs and ensures DNA elimination in Tetrahymena. *RNA*.

[19] Li J., Yang Z., Yu B., Liu J., Chen X. (2005). Methylation protects miRNAs and siRNAs from a 3′-end uridylation activity in Arabidopsis. *Current Biology*.

[20] Mullen T. E., Marzluff W. F. (2008). Degradation of histone mRNA requires oligouridylation followed by decapping and simultaneous degradation of the mRNA both 5′ to 3′ and 3′ to 5′. *Genes and Development*.

[21] Schmidt M.-J., West S., Norbury C. J. (2011). The human cytoplasmic RNA terminal U-transferase ZCCHC11 targets histone mRNAs for degradation. *RNA*.

[22] Yu B., Yang Z., Li J. (2005). Methylation as a crucial step in plant microRNA biogenesis. *Science*.

[23] Parker R., Song H. (2004). The enzymes and control of eukaryotic mRNA turnover. *Nature Structural and Molecular Biology*.

[24] Wilkie G. S., Dickson K. S., Gray N. K. (2003). Regulation of mRNA translation by 5′- and 3′-UTR-binding factors. *Trends in Biochemical Sciences*.

[25] Babendure J. R., Babendure J. L., Ding J.-H., Tsien R. Y. (2006). Control of mammalian translation by mRNA structure near caps. *RNA*.

[26] Bashirullah A., Cooperstock R. L., Lipshitz H. D. (2001). Spatial and temporal control of RNA stability. *Proceedings of the National Academy of Sciences of the United States of America*.

[27] Conne B., Stutz A., Vassalli J.-D. (2000). The 3′ untranslated region of messenger RNA: a molecular ‘hotspot’ for pathology?. *Nature Medicine*.

[28] Mayr C., Bartel D. P. (2009). Widespread shortening of 3′UTRs by alternative cleavage and polyadenylation activates oncogenes in cancer cells. *Cell*.

[29] van der Velden A. W., Thomas A. A. M. (1999). The role of the 5′ untranslated region of an mRNA in translation regulation during development. *The International Journal of Biochemistry & Cell Biology*.

[30] Benne R., van den Burg J., Brakenhoff J. P. J., Sloof P., van Boom J. H., Tromp M. C. (1986). Major transcript of the frameshifted coxll gene from trypanosome mitochondria contains four nucleotides that are not encoded in the DNA. *Cell*.

[31] Decker C. J., Parker R. (1993). A turnover pathway for both stable and unstable mRNAs in yeast: evidence for a requirement for deadenylation. *Genes and Development*.

[32] Dunckley T., Parker R. (1999). The DCP2 protein is required for mRNA decapping in *Saccharomyces cerevisiae* and contains a functional MutT motif. *The EMBO Journal*.

[33] Lykke-Andersen J. (2002). Identification of a human decapping complex associated with hUpf proteins in nonsense-mediated decay. *Molecular and Cellular Biology*.

[34] Seiwert S. D., Heidmann S., Stuart K. (1996). Direct visualization of uridylate deletion in vitro suggests a mechanism for kinetoplastid RNA editing. *Cell*.

[35] Shyu A.-B., Belasco J. G., Greenberg M. E. (1991). Two distinct destabilizing elements in the C-fos message trigger deadenylation as a first step in rapid mRNA decay. *Genes and Development*.

[36] Schoenberg D. R., Maquat L. E. (2012). Regulation of cytoplasmic mRNA decay. *Nature Reviews Genetics*.

[37] Carballo E., Lai W. S., Blackshear P. J. (1998). Feedback inhibition of macrophage tumor necrosis factor-*α* production by tristetraprolin. *Science*.

[38] Lai W. S., Carballo E., Thorn J. M., Kennington E. A., Blackshear P. J. (2000). Interactions of CCCH zinc finger proteins with mRNA. Binding of tristetraprolin-related zinc finger proteins to AU-rich elements and destabilization of mRNA. *The Journal of Biological Chemistry*.

[39] Hsu C. L., Stevens A. (1993). Yeast cells lacking 5′ → 3′ exoribonuclease 1 contain mRNA species that are poly(A) deficient and partially lack the 5′ cap structure. *Molecular and Cellular Biology*.

[40] van Dijk E., Cougot N., Meyer S., Babajko S., Wahle E., Séraphin B. (2002). Human Dcp2: a catalytically active mRNA decapping enzyme located in specific cytoplasmic structures. *The EMBO Journal*.

[41] Anderson J. S. J., Parker R. (1998). The 3′ to 5′ degradation of yeast mRNAs is a general mechanism for mRNA turnover that requires the SK12 DEVH box protein and 3′ to 5′ exonucleases of the exosome complex. *EMBO Journal*.

[42] Wang Z., Kiledjian M. (2001). Functional link between the mammalian exosome and mRNA decapping. *Cell*.

[43] Canellakis E. S. (1957). Incorporation of radioactive uridine-5′-monophosphate into ribonucleic acid by soluble mammalian enzymes. *Biochimica et Biophysica Acta*.

[44] Wilkie N. M., Smellie R. M. (1968). Chain extension of ribonucleic acid by enzymes from rat liver cytoplasm. *Biochemical Journal*.

[45] Wilkie N. M., Smellie R. M. (1968). Polyribonucleotide synthesis by subfractions of microsomes from rat liver. *Biochemical Journal*.

[46] Aphasizhev R., Aphasizheva I., Simpson L. (2003). A tale of two TUTases. *Proceedings of the National Academy of Sciences of the United States of America*.

[47] Aphasizheva I., Maslov D., Wang X., Huang L., Aphasizhev R. (2011). Pentatricopeptide repeat proteins stimulate mRNA adenylation/uridylation to activate mitochondrial translation in trypanosomes. *Molecular Cell*.

[48] Wang S.-W., Toda T., MacCallum R., Harris A. L., Norbury C. (2000). Cid1, a fission yeast protein required for S-M checkpoint control when DNA polymerase *δ* or *ε* is inactivated. *Molecular and Cellular Biology*.

[49] Lim J., Ha M., Chang H. (2014). Uridylation by TUT4 and TUT7 marks mRNA for degradation. *Cell*.

[50] Steltz T. A. (1998). A mechanism for all polymerases. *Nature*.

[51] Deng J., Ernst N. L., Turley S., Stuart K. D., Hol W. G. J. (2005). Structural basis for UTP specificity of RNA editing TUTases from *Trypanosoma brucei*. *The EMBO Journal*.

[52] Stagno J., Aphasizheva I., Bruystens J., Luecke Hartmut H., Aphasizhev R. (2010). Structure of the mitochondrial editosome-like complex associated TUTase 1 reveals divergent mechanisms of UTP selection and domain organization. *Journal of Molecular Biology*.

[53] Munoz-Tello P., Gabus C., Thore S. (2012). Functional implications from the Cid1 poly(U) polymerase crystal structure. *Structure*.

[54] Chang H., Lim J., Ha M., Kim V. N. (2014). TAIL-seq: genome-wide determination of poly(A) tail length and 3′ end modifications. *Molecular Cell*.

[55] Martin G., Keller W. (1996). Mutational analysis of mammalian poly(A) polymerase identifies a region for primer binding and catalytic domain, homologous to the family X polymerases, and to other nucleotidyltransferases. *The EMBO Journal*.

[56] Zhelkovsky A. M., Kessler M. M., Moore C. L. (1995). Structure-function relationships in the *Saccharomyces cerevisiae* poly(A) polymerase. Identification of a novel RNA binding site and a domain that interacts with specificity factor(s). *The Journal of Biological Chemistry*.

[57] Chang H.-M., Martinez N. J., Thornton J. E., Hagan J. P., Nguyen K. D., Gregory R. I. (2012). Trim71 cooperates with microRNAs to repress Cdkn1a expression and promote embryonic stem cell proliferation. *Nature Communications*.

[58] Heo I., Joo C., Kim Y.-K. (2009). TUT4 in concert with Lin28 suppresses microRNA biogenesis through pre-microRNA uridylation. *Cell*.

[59] Yu J., Vodyanik M. A., Smuga-Otto K. (2007). Induced pluripotent stem cell lines derived from human somatic cells. *Science*.

[60] Aravind L., Koonin E. V. (1999). DNA polymerase *β*-like nucleotidyltransferase superfamily: identification of three new families, classification and evolutionary history. *Nucleic Acids Research*.

[61] Betat H., Rammelt C., Martin G., Mörl M. (2004). Exchange of regions between bacterial poly(A) polymerase and the CCA-adding enzyme generates altered specificities. *Molecular Cell*.

[62] Rissland O. S., Mikulasova A., Norbury C. J. (2007). Efficient RNA polyuridylation by noncanonical poly(A) polymerases. *Molecular and Cellular Biology*.

[63] Aphasizhev R., Aphasizheva I. (2011). Uridine insertion/deletion editing in trypanosomes: a playground for RNA-guided information transfer. *Wiley Interdisciplinary Reviews: RNA*.

[64] Trippe R., Guschina E., Hossbach M., Urlaub H., Lührmann R., Benecke B.-J. (2006). Identification, cloning, and functional analysis of the human U6 snRNA-specific terminal uridylyl transferase. *RNA*.

[65] Aphasizhev R., Aphasizheva I. (2008). Terminal RNA uridylyltransferases of trypanosomes. *Biochimica et Biophysica Acta—Gene Regulatory Mechanisms*.

[66] Aphasizhev R., Sbicego S., Peris M. (2002). Trypanosome mitochondrial 3′ terminal uridylyl transferase (TUTase): the key enzyme in U-insertion/deletion RNA editing. *Cell*.

[67] Rusché L. N., Cruz-Reyes J., Piller K. J., Sollner-Webb B. (1997). Purification of a functional enzymatic editing complex from *Trypanosoma brucei* mitochondria. *EMBO Journal*.

[68] Stuart K. D., Schnaufer A., Ernst N. L., Panigrahi A. K. (2005). Complex management: RNA editing in trypanosomes. *Trends in Biochemical Sciences*.

[69] Panigrahi A. K., Schnaufer A., Ernst N. L. (2003). Identification of novel components of *Trypanosoma brucei* editosomes. *RNA*.

[70] Wang B., Ernst N. L., Palazzo S. S., Panigrahi A. K., Salavati R., Stuart K. (2003). TbMP44 is essential for RNA editing and structural integrity of the editosome in *Trypanosoma brucei*. *Eukaryotic Cell*.

[71] Ernst N. L., Panicucci B., Igo R. P., Panigrahi A. K., Salavati R., Stuart K. (2003). TbMP57 is a 3′ terminal uridylyl transferase (TUTase) of the *Trypanosoma brucei* editosome. *Molecular Cell*.

[72] Abelson J. (1979). RNA processing and the intervening sequence problem. *Annual Review of Biochemistry*.

[73] Ibrahim F., Rymarquis L. A., Kim E.-J. (2010). Uridylation of mature miRNAs and siRNAs by the MUT68 nucleotidyltransferase promotes their degradation in *Chlamydomonas*. *Proceedings of the National Academy of Sciences of the United States of America*.

[74] van Wolfswinkel J. C., Claycomb J. M., Batista P. J., Mello C. C., Berezikov E., Ketting R. F. (2009). CDE-1 affects chromosome segregation through uridylation of CSR-1-bound siRNAs. *Cell*.

[75] Zhao Y. Y., Yu Y., Zhai J. (2012). The *Arabidopsis* nucleotidyl transferase HESO1 uridylates unmethylated small RNAs to trigger their degradation. *Current Biology*.

[76] Trippe R., Sandrock B., Benecke B.-J. (1998). A highly specific terminal uridylyl transferase modifies the 3′-end of U6 small nuclear RNA. *Nucleic Acids Research*.

[77] Lund E., Dahlberg J. E. (1992). Cyclic 2′-3′-phosphates and nontemplated nucleotides at the 3′ end of spliceosomal U6 small nuclear RNA's. *Science*.

[78] Rinke J., Steitz J. A. (1985). Association of the lupus antigen La with a subset of U6 snRNA molecules. *Nucleic Acids Research*.

[79] Chen Y., Sinha K., Perumal K., Reddy R. (2000). Effect of 3′ terminal adenylic acid residue on the uridylation of human small RNAs in vitro and in frog oocytes. *RNA*.

[80] Zimmer S. L., Schein A., Zipor G., Stern D. B., Schuster G. (2009). Polyadenylation in Arabidopsis and *Chlamydomonas* organelles: the input of nucleotidyltransferases, poly(A) polymerases and polynucleotide phosphorylase. *Plant Journal*.

[81] Raynal L. C., Carpousis A. J. (1999). Poly(A) polymerase I of *Escherichia coli*: characterization of the catalytic domain, an RNA binding site and regions for the interaction with proteins involved in mRNA degradation. *Molecular Microbiology*.

[82] Blum E., Carpousis A. J., Higgins C. F. (1999). Polyadenylation promotes degradation of 3′-structured RNA by the *Escherichia coli* mRNA degradosome in vitro. *The Journal of Biological Chemistry*.

[83] Lisitsky I., Klaff P., Schuster G. (1996). Addition of destabilizing poly(A)-rich sequences to endonuclease cleavage sites during the degradation of chloroplast mRNA. *Proceedings of the National Academy of Sciences of the United States of America*.

[84] Lisitsky I., Kotler A., Schuster G. (1997). The mechanism of preferential degradation of polyadenylated RNA in the chloroplast: the exoribonuclease 100RNP/polynucleotide phosphorylase displays high binding affinity for poly(A) sequence. *The Journal of Biological Chemistry*.

[85] Weng J., Aphasizheva I., Etheridge R. D. (2008). Guide RNA-binding complex from mitochondria of trypanosomatids. *Molecular Cell*.

[86] Borowski L. S., Szczesny R. J., Brzezniak L. K., Stepien P. P. (2010). RNA turnover in human mitochondria: more questions than answers?. *Biochimica et Biophysica Acta*.

[87] Slomovic S., Laufer D., Geiger D., Schuster G. (2005). Polyadenylation and degradation of human mitochondrial RNA: the prokaryotic past leaves its mark. *Molecular and Cellular Biology*.

[88] Slomovic S., Schuster G. (2008). Stable PNPase RNAi silencing: its effect on the processing and adenylation of human mitochondrial RNA. *RNA*.

[89] Szczesny R. J., Borowski L. S., Brzezniak L. K. (2010). Human mitochondrial RNA turnover caught in flagranti: involvement of hSuv3p helicase in RNA surveillance. *Nucleic Acids Research*.

[90] Song M.-G., Kiledjian M. (2007). 3′ terminal oligo U-tract-mediated stimulation of decapping. *RNA*.

[91] Osley M. A. (1991). The regulation of histone synthesis in the cell cycle. *Annual Review of Biochemistry*.

[92] Battle D. J., Doudna J. A. (2001). The stem-loop binding protein forms a highly stable and specific complex with the 3′ stem-loop of histone mRNAs. *RNA*.

[93] Gallie D. R., Lewis N. J., Marzluff W. F. (1996). The histone 3′-terminal stem-loop is necessary for translation in Chinese hamster ovary cells. *Nucleic Acids Research*.

[94] Pandey N. B., Marzluff W. F. (1987). The stem-loop structure at the 3′ end of histone mRNA is necessary and sufficient for regulation of histone mRNA stability. *Molecular and Cellular Biology*.

[95] Hoefig K. P., Rath N., Heinz G. A. (2013). Eri1 degrades the stem-loop of oligouridylated histone mRNAs to induce replication-dependent decay. *Nature Structural and Molecular Biology*.

[96] Heo I., Ha M., Lim J. (2012). Mono-uridylation of pre-microRNA as a key step in the biogenesis of group II let-7 microRNAs. *Cell*.

[97] Ibrahim F., Rohr J., Jeong W.-J., Hesson J., Cerutti H. (2006). Untemplated oligoadenylation promotes degradation of RISC-cleaved transcripts. *Science*.

[98] Thornton J. E., Chang H.-M., Piskounova E., Gregory R. I. (2012). Lin28-mediated control of let-7 microRNA expression by alternative TUTases Zcchc11 (TUT4) and Zcchc6 (TUT7). *RNA*.

[99] Choi Y. S., Patena W., Leavitt A. D., Mcmanus M. T. (2012). Widespread RNA 3′-end oligouridylation in mammals. *RNA*.

[100] Landgraf P., Rusu M., Sheridan R. (2007). A mammalian microRNA expression atlas based on small RNA library sequencing. *Cell*.

[101] Morin R. D., O'Connor M. D., Griffith M. (2008). Application of massively parallel sequencing to microRNA profiling and discovery in human embryonic stem cells. *Genome Research*.

[102] Newman M. A., Mani V., Hammond S. M. (2011). Deep sequencing of microRNA precursors reveals extensive 3′ end modification. *RNA*.

[103] Hagan J. P., Piskounova E., Gregory R. I. (2009). Lin28 recruits the TUTase Zcchc11 to inhibit let-7 maturation in mouse embryonic stem cells. *Nature Structural and Molecular Biology*.

[104] Lehrbach N. J., Armisen J., Lightfoot H. L. (2009). LIN-28 and the poly(U) polymerase PUP-2 regulate let-7 microRNA processing in *Caenorhabditis elegans*. *Nature Structural and Molecular Biology*.

[105] Loughlin F. E., Gebert L. F. R., Towbin H., Brunschweiger A., Hall J., Allain F. H.-T. (2012). Structural basis of pre-let-7 miRNA recognition by the zinc knuckles of pluripotency factor Lin28. *Nature Structural and Molecular Biology*.

[106] Nam Y., Chen C., Gregory R. I., Chou J. J., Sliz P. (2011). Molecular basis for interaction of let-7 MicroRNAs with Lin28. *Cell*.

[107] Jones M. R., Quinton L. J., Blahna M. T. (2009). Zcchc11-dependent uridylation of microRNA directs cytokine expression. *Nature Cell Biology*.

[108] Lubas M., Damgaard C. K., Tomecki R., Cysewski D., Jensen T. H., Dziembowski A. (2013). Exonuclease hDIS3L2 specifies an exosome-independent 3′-5′ degradation pathway of human cytoplasmic mRNA. *The EMBO Journal*.

[109] Malecki M., Viegas S. C., Carneiro T. (2013). The exoribonuclease Dis3L2 defines a novel eukaryotic RNA degradation pathway. *The EMBO Journal*.

[110] Ren G., Chen X., Yu B. (2012). Uridylation of miRNAs by hen1 suppressor1 in *Arabidopsis*. *Current Biology*.

[111] Claycomb J. M., Batista P. J., Pang K. M. (2009). The Argonaute CSR-1 and its 22G-RNA cofactors are required for holocentric chromosome segregation. *Cell*.

[112] Gu W., Shirayama M., Conte D. (2009). Distinct argonaute-mediated 22G-RNA pathways direct genome surveillance in the *C. elegans* germline. *Molecular Cell*.

[113] Ren G., Xie M., Zhang S., Vinovskis C., Chen X., Yu B. (2014). Methylation protects microRNAs from an AGO1-associated activity that uridylates 5' RNA fragments generated by AGO1 cleavage. *Proceedings of the National Academy of Sciences of the United States of America*.

[114] Sement F. M., Ferrier E., Zuber H. (2013). Uridylation prevents 3′ trimming of oligoadenylated mRNAs. *Nucleic Acids Research*.

[115] Minoda Y., Saeki K., Aki D. (2006). A novel Zinc finger protein, ZCCHC11, interacts with TIFA and modulates TLR signaling. *Biochemical and Biophysical Research Communications*.

[116] Piskounova E., Polytarchou C., Thornton J. E. (2011). Lin28A and Lin28B inhibit let-7 MicroRNA biogenesis by distinct mechanisms. *Cell*.

[117] Viswanathan S. R., Powers J. T., Einhorn W. (2009). Lin28 promotes transformation and is associated with advanced human malignancies. *Nature Genetics*.

[118] Chang H.-M., Triboulet R., Thornton J. E., Gregory R. I. (2013). A role for the Perlman syndrome exonuclease Dis3l2 in the Lin28-let-7 pathway. *Nature*.

[119] Clamer M., Höfler L., Mikhailova E., Viero G., Bayley H. (2014). Detection of 3′-end RNA uridylation with a protein nanopore. *ACS Nano*.

